# Microsatellite instability is inversely associated with type 2 diabetes mellitus in colorectal cancer

**DOI:** 10.1371/journal.pone.0215513

**Published:** 2019-04-19

**Authors:** Yujiro Nakayama, Takeru Iijima, Rika Wakaume, Keiichi Takahashi, Hiroshi Matsumoto, Daisuke Nakano, Michiko Miyaki, Tatsuro Yamaguchi

**Affiliations:** 1 Department of Surgery, Tokyo Metropolitan Cancer and Infectious diseases Center Komagome Hospital, Tokyo, Japan; 2 Department of Minimally Invasive Surgical and Medical Oncology, Fukushima Medical University, Fukushima, Japan; 3 Department of Surgery, Southern Tohoku General Hospital, Fukushima, Japan; 4 Hereditary Tumor Research Project, Tokyo Metropolitan Cancer and Infectious diseases Center Komagome Hospital, Tokyo, Japan; University of Newcastle, AUSTRALIA

## Abstract

**Background:**

Microsatellite instability (MSI) is a clonal change in the number of repeated DNA nucleotide units in microsatellites. High-frequency MSI (MSI-H) colorectal cancers (CRCs) are known to have different clinicopathological features compared with microsatellite stable (MSS) CRCs. In addition, previous studies have shown that type2 diabetes mellitus (T2DM) is a risk factor for malignant tumors including CRCs. The aim of this study was to investigate the relationship between T2DM and MSI-H colorectal cancer.

**Methods:**

The study design is a single center, cross-sectional study. Data from a series of 936 patients with CRCs were collected and MSI status was assessed.

**Results:**

In total, 29 (3.1%) and 907 (96.9%) tumors were classified as having MSI-H and low-frequency microsatellite instability or being MSS (MSS), respectively. Of the 936 patients, 275 (29.6%) were associated with T2DM. One (3.4%) of the 29 MSI-H patients and 274 (30.2%) of the 907 MSS patients had T2DM. Thus, the incidence of T2DM was significantly less frequent in MSI-H compared with MSS patients (Fisher’s exact test: p = 0.0007).

**Conclusions:**

We conclude that MSS tumors are significantly more common than MSI-H tumors among individuals with T2DM.

## Introduction

Colorectal cancer (CRC) is one of the most common solid tumors, and details associated with its carcinogenesis have been intensively studied. Some colorectal cancers are microsatellite stable (MSS). The typical development of MSS tumors proceeds stepwise by the inactivation of increasing numbers of tumor suppressor genes, including the *APC* and *p53* genes, through mutation as well as loss of heterozygosity (LOH) and the activation of oncogenes such as *KRAS* [1, 2].

On the other hand, microsatellite instability (MSI) is a phenotype resulting from a defect in mismatch repair genes such as *MSH2* [3, 4], *MLH1* [4], and *MSH6* [5, 6]. No MSI tumor shows LOH at these tumor suppressor genes [7], and target genes for frameshift mutations in CRCs are different from those in MSS tumors. Well-known target genes for MSI tumors include *TGFβRII* [8], *IGFIIR* [9], and *BAX* [10]. Testing colorectal cancers for MSI is an effective method of screening for Lynch syndrome because approximately 90% of Lynch syndrome tumors have high microsatellite instability (MSI-H) [11]. Although MSI is also observed in sporadic CRC, CRCs with MSI-H, irrespective of whether they are hereditary or not, have important therapeutic and diagnostic characteristics. CRCs with MSI-H are generally associated with a better prognosis [12], but their prognosis is less favorable with 5-FU based chemotherapy [13]; in addition, these tumors have less metastasis, and are more likely to be a right sided or metachronous multiple CRCs [12, 14].

Type 2 diabetes mellitus (T2DM) is associated with malignant tumors, including CRC [15]. It has been reported that CRC incidences in diabetic patients were 1.27–1.40 in colon and 1.19–1.36 in rectum [16–18]. Jiang Y et al. reported that relative risk of CRC incidence for T2DM were 1.27 (95% CI: 1.21–1.34) by meta-analysis of cohort studies [19]. However, the relationship between microsatellite instability of CRC and T2DM has not been clear yet. In this study, we investigated a relationship between T2DM and MSI in CRC.

## Materials and methods

### Patients

This study is a single center, retrospective cross-sectional study. We selected 936 consecutive colorectal cancer patients who underwent surgical resection at the Tokyo Metropolitan Cancer and Infectious diseases Center Komagome Hospital from January 2008 to January 2014 after obtaining their informed consent. The study was performed after approval of the Tokyo Metropolitan Cancer and Infectious Diseases Center Komagome Hospital Ethical Committee (ID: 612). Patients with inflammatory bowel disease or a known history of familial adenomatous polyposis and Lynch syndrome were excluded. If the patient had two or more colorectal tumors resected, then the tumor that was most advanced was selected for analysis.

We defined patients with T2DM as those who had already been diagnosed with T2DM or those who had a high concentration of glycated hemoglobin (HbA1c > 6.5%) which was measured before the first colorectal surgery. The criteria for T2DM diagnosis depends on Standards of Medical Care in Diabetes 2018 by American Diabetes Association [20].

Body weight (kg) and height (m) were measured immediately before surgery. The body mass index (BMI) was calculated as weight in kilograms divided by the square of the height in meters (kg/m^2^).

#### Microsatellite instability analysis and mutation analysis

Colorectal cancers and corresponding normal tissues were obtained with informed consent and were stored at −80°C immediately after resection. Genomic DNA samples were extracted using the QIAamp DNA mini kit (QIAGEN, Valencia, CA,USA). Methods to determine microsatellite instability and *KRAS* and *BRAF* mutations were describe previously. Briefly, polymerase chain reaction (PCR) was performed to amplify at least five repetitive sequence loci from the tumor and normal tissue samples: BAT25, BAT26, D2S123, D5S346, and D17S250. Microsatellite instability status was defined as MSI-H (2–5 of the 5 markers used were unstable) and MSS (none or only 1 of the 5 markers was unstable), as described in the National Cancer Institute guidelines for MSI testing (22). All samples were analyzed to identify any *BRAF* (V600E) and *KRAS* (codons 12 and 13) mutations by direct sequencing.

### Statistical analysis

The Fisher’s exact test was used to evaluate the relationship between two discrete and dichotomous variables. The analysis of association between categorical variables was performed using logistic regression method. An optimal cut-off value for predicting MSI status was analyzed using receiver operating characteristic (ROC) curves. Areas under the curve (AUC) were also calculated. All statistical tests were 2-sided, and P values of ≤0.05 were considered to indicate statistical significance. All statistical analyses were performed with EZR (Saitama Medical Center, Jichi Medical University, Japan), which is a graphical-user interface for R (The R Foundation for Statistical Computing, Vienna, Austria, version 3.5.1). This interface is a modified version of R commander (version 2.5–1) that includes statistical functions that are frequently used in biostatistics.

## Results

Total of 936 CRC patients were enrolled during study period in this study. All patients underwent surgical resection of the primary tumor and the diagnosis of adenocarcinoma was made pathologically. None received neo-adjuvant chemotherapy or radiotherapy. The clinical and pathological characteristics of the patients are shown in Table 1. Of the 936 colorectal cancers, 29 (3.1%) and 907 (96.9%) tumors were classified as MSI-H and MSS, respectively. Significant differences between MSI-H and MSS cancers were observed with respect to sex (p = 0.022), age (p = 0.007), tumor location (p < 0.0001) and histology (p < 0.0001); whereas, there were no significant differences with respect to UICC classification (p = 0.57). One hundred seventy-five (18.7%) patients had T2DM and no patient had type 1 diabetes mellitus. The incidence of T2DM was significantly less frequent in MSI-H than MSS patients (p = 0.0007). However, there was no significant difference in BMI between MSI-H and MSS patients (p = 0.65). Of the 936 CRC patients, 277 (29.6%) had *KRAS* mutation and 49 (5.2%) had *BRAF* mutation. None of the 29 MSI-H patients were a *KRAS* mutation, whereas 277 (30.5%) of the 907 MSS patients had a *KRAS* mutation, and frequency of *KRAS* mutation was significantly different between MSI-H and MSS (p < 0.0001). Inversely, frequency of BRAF mutation was significantly high in MSI-H patients than in MSS patients (p < 0.0001). According to logistic regression analysis, age, tumor location, histology and T2DM status were independent factors in MSI-H tumor (Table 2).

**Table 1:** Clinicopathological characteristics of the patients.

Features	MSI-H	MSS	Total	P value
Total	29	907	936	
Gender				0.022
Male	10	514	524	
Female	19	393	412	
Age (years old)				0.007
≦69	9	516	525	
≧70	20	391	411	
Location				<0.0001
Right ^a^	24	220	244	
Left ^b^	5	687	692	
Histology				<0.0001
wel/mod ^c^	16	845	861	
muc/por ^d^	13	62	75	
UICC classification				0.57
Stage 0- II	15	411	426	
Stage III- IV	14	496	510	
Type 2 diabetes mellitus				0.0007
−	28	633	661	
+	1	274	275	
Body mass index				0.65
<25.00 kg/m^2^	21	698	719	
≧25.00 kg/m^2^	8	209	217	
*KRAS* gene				<0.0001
Wild type	29	630	659	
Mutant type	0	277	277	
*BRAF* gene				<0.0001
Wild type	7	880	887	
Mutant type	22	27	49	

^a^ Right: Cecum, Ascending colon, and Transverse colon

^b^ Left: Descending colon, Sigmoid colon, Rectosigmoid colon, and Rectum

^c^ wel: well differentiated adenocarcinoma; mod: moderately differentiated adenocarcinoma

^d^ muc: mucinous carcinoma; por: poorly differentiated adenocarcinoma

**Table 2:** Multivariate analysis of factors predicting MSI-H tumor.

	Odds ratio	95% confidence interval	P value
Gender (male: female)	2.04	0.86–4.87	0.11
Age (≦69: 70≦)	2.01	0.84–4.85	0.12
Location (Right ^a^: Left ^b^)	0.10	0.04–0.28	< 0.0001
Histology (wel/mod ^c^: muc/por ^d^)	7.76	3.26–18.50	< 0.0001
Type 2 diabetes mellitus (-: +)	0.11	0.01―0.82	0.032

^a^ Right: Cecum, Ascending colon, and Transverse colon

^b^ Left: Descending colon, Sigmoid colon, Rectosigmoid colon, and Rectum

^c^ wel: well differentiated adenocarcinoma; mod: moderately differentiated adenocarcinoma

^d^ muc: mucinous carcinoma; por: poorly differentiated adenocarcinoma

Table 3 showed clinicopathological features according to T2DM status. T2DM+ was significantly more frequent in male patients, elderly patients and obesity patients; however, no correlation was seen between T2DM+ and tumor location, histology or UICC classification. *BRAF* gene mutation was significantly more frequent in T2DM- patients, while *KRAS* gene mutation was not correlated with T2DM status.

**Table 3:** Clinicopathological characteristics of the patients.

Features	T2DM-	T2DM+	Total	P value
Total	661	275	936	
Gender				<0.0001
Male	343	181	524	
Female	318	94	412	
Age (years old)				0.021
≦69	387	138	525	
≧70	274	137	411	
Location				0.46
Right ^a^	177	67	244	
Left ^b^	484	208	692	
Histology				0.16
wel/mod ^c^	602	259	861	
muc/por ^d^	59	16	75	
UICC classification				0.94
Stage 0- II	300	126	426	
Stage III- IV	361	149	510	
MSI status				0.0007
MSI-H	28	1	29	
MSS	633	274	907	
Body mass index				0.0005
<25.00 kg/m^2^	529	190	719	
≧25.00 kg/m^2^	132	85	217	
*KRAS* gene				0.48
Wild type	470	189	659	
Mutant type	191	86	277	
*BRAF* gene				0.0057
Wild type	618	269	887	
Mutant type	43	6	49	

^a^ Right: Cecum, Ascending colon, and Transverse colon

^b^ Left: Descending colon, Sigmoid colon, Rectosigmoid colon, and Rectum

^c^ wel: well differentiated adenocarcinoma; mod: moderately differentiated adenocarcinoma

^d^ muc: mucinous carcinoma; por: poorly differentiated adenocarcinoma

## Discussion

Our study findings indicated that T2DM is significantly less common among MSI-H patients compared with MSS patients. This is the first report to indicate that T2DM may be associated with MSS CRC. There have been no previous reports of a relationship between T2DM and MSI status in CRC, even though it is well known that the incidence of malignant tumors is increased in patients with T2DM [7, 21].

Our study revealed the frequency of MSI-H tumors to be 3.1%, which reported as well as in previous studies in Asian countries is lower than that reported in Western countries [22, 23]. Asaka et al reported on the frequency of MSI in 940 Japanese CRC patients and found that 5.9% were MSI-H and 94.1% were MSS/MSI-L [24]. The incidence of rectal cancer is higher in Japanese individuals (approximately 40% of CRCs) compared with individuals from Western countries (approximately 20% of CRCs), which would reflect a lower rate of MSI-H CRC because rectal cancer is less likely to show MSI-H than colon cancer. In addition, as we reported previously, MSI-H is less frequent even in right colon cancer in Japanese individuals than in Western. [25]. Race may thus affect MSI status.

T2DM has been shown to be a risk factor for malignant tumors including CRC [15]. T2DM and CRC are major causes of morbidity and mortality in the United States, Western countries, and increasingly in Japan [26, 27]. Dietary and lifestyle risk factors for developing insulin resistance and T2DM, such as a Western diet, physical inactivity, and obesity, have also been linked to an increased risk of CRC [28–30]. Furthermore, an association between metabolic syndrome and CRC is now supported by a large number of epidemiological studies [16, 17, 31–33]. In this study, the frequency of obesity was almost identical between the MSS and MSI-H groups, and thus, we were able to compare the incidence of T2DM as an independent risk factor for colorectal cancer.

Previous reports have shown that in patients who suffer from T2DM, hyperinsulinemia, or factors related to insulin resistance, such as hyperglycemia or hypertriglyceridemia, are associated with colorectal carcinogenesis [16]. IGF-1, which is suggested to stimulate cell proliferation by its activation, is reported to be associated with CRC both epidemiologically and experimentary [34]. Moreover, biologic interactions among insulin, IGF-1, and IGFBPs may increase the risk of CRC through diet and associated factors, including Wnt pathway and PI3K/Akt pathway [35–40].

The current study had some limitations as follows: (1) selection bias caused by retrospective nature of the study; (2) a single-center study; (3) our study revealed that T2DM was associated with MSS colorectal cancer; however, we could not provide the mechanism. Nonetheless, considering that there are only a few publications on association with T2DM and MSS colorectal cancer, we believe that our findings will help researchers and physicians clarify the nature of colorectal cancer. And, we also know that further studies are required to overcome these limitations.

### Conclusions

We conclude that MSS tumors are significantly more common than MSI-H tumors among individuals with T2DM.

## Supporting information

S1 File NAKAYAMA_PONE-D-18-34914new. Supplemental data.
(XLSX)
